# Preliminary Study for Inspecting Moisture Content, Dry Matter Content, and Firmness Parameters of Two Date Cultivars Using an NIR Hyperspectral Imaging System

**DOI:** 10.3389/fbioe.2021.720630

**Published:** 2021-10-21

**Authors:** Ayman Ibrahim, Abdulrahman Alghannam, Ayman Eissa, Ferenc Firtha, Timea Kaszab, Zoltan Kovacs, Lajos Helyes

**Affiliations:** ^1^ Agricultural Engineering Research Institute (AEnRI), Agricultural Research Center (ARC), Giza, Egypt; ^2^ Department of Agricultural Systems Engineering, College of Agricultural and Food Sciences, King Faisal University, Al-Hassa, Saudi Arabia; ^3^ Department of Agricultural Engineering, Faculty of Agriculture, Menoufia University, Shebin El Koum, Egypt; ^4^ Department of Measurements and Process Control, Institute of Food Science and Technology, Hungarian University of Agriculture and Life Sciences, Budapest, Hungary; ^5^ Horticultural institute, Hungarian University of Agriculture and Life Sciences, Gödöllő, Hungary

**Keywords:** date fruits, quality, hyperspectral imaging, texture, partial least squares regression

## Abstract

The assessment and assurance of the quality attributes of dates is a key factor in increasing the competitiveness and consumer acceptance of this fruit. The increasing demand for date fruits requires a rapid and automated method for monitoring and analyzing the quality attributes of date fruits to replace the conventional methods used by inspection which limits the production and involves human errors. Moisture content (MC), dry matter content (DMC), and firmness (F) are three important quality attributes for two date cultivars (Khalas and Sukkari) that have been inspected using the hyperspectral imaging (HSI) technique based on the reflectance mode. Images of intact date fruits at the maturity stage Tamr were obtained within the wavelength range of 950–1750 nm. Monitoring and assessment of MC, DMC, and F [first maximum rupture force (MF, N)] were performed using a partial least squares regression model. Accurate prediction models were attained. The results highlight that the coefficients of determination (R^2^_Prediction_) are estimated to be 0.91 and 0.89 for MC, DMC, and F (N) with the lowest values of the standard error of prediction (SEP) equal to 0.82, 0.81 (%), and 4.12 (N), respectively, and the residual predictive deviation (RPD) values were 3.65, 3.69, and 3.42 for MC, DMC, and F (N), respectively. The results obtained from this preliminary study indicate the great potential of applying HSI for the assessment of physical, chemical, and sensory quality attributes of date fruits overall in the five maturity stages.

## Introduction

In general, fruit quality is considered a basic requirement for consumers seeking good appearance, acceptable firmness, a nutritional value, and fruits freedom from external and internal defects and injuries. The date fruit is one of the most important fruits with a high nutritional value, which occupies a prominent position on the world fruit map. According to FAO (2019), Egypt, the Kingdom of Saudi Arabia, and Iran are the leading date-producing and exporting countries. Globally, date palm production covers an area of nearly 1.09 million ha with a total production of about 8.53 million tons. However, the world date production was valued at about US$ 8.4 billion, with global trade amounting to about US$ 1.2 billion, providing a major source of export revenues as well as livelihoods and income for millions of rural smallholders FAO (2018). The ripening of date fruits is classified into five maturity stages (Hababouk, Kimri, Khalal, Rutab, and Tamr). However, the quality inspection tasks during the harvest and post-harvest operations are based on physical properties such as color, moisture content, and firmness Kader and Awad (2009); Gross et al. (2016). The ripening of dates in all maturity stages relies on color as one of the most important quality attributes of dates, in addition to the levels of moisture and sugar content Farahnaky and Afshari-Jouybari (2010). In this regard, Singh et al. (2012) and Rahman and Al-Farsi (2005) cited that date fruits are consumed as fresh fruits at the Khalal and Rutab maturity stages, while it is consumed mainly in the dry form in the Tamr maturity stage when dates are firm, have a lower moisture content, and are sweeter. Furthermore, Singh et al. (2013), classified dates available on the market into three different categories: hardresilient, soft-springy, and firm-adhesive. These categories are based on physicochemical properties determined using instrumented texture profile analysis (TPA) testing. Ghnimi et al. (2017) announced that date fruits at full maturity (Tamr stage) are mainly composed of sugars (60–80%), moisture (10–30%), dietary fiber (5–12%), phenolic compounds (up to 4%), and other minor elementals based on a fresh weight basis. The textural properties of the most popular eight Saudi date cultivars at the Tamr maturity stage were measured by Alhamdan et al. (2016). It was found that the hardness values were 65.60, 186.59, 38.04, 68.72, 140.31, 394.65, 55.97, and 79.45 N in light of differences in the moisture content values of 6.92, 11.24, 8.96, 7.75, 12.56, 7.93, 11.71, and 11.32% for Barhi, Khudari, Khlas, Serrie, Sukkari, Suffri, Saqie, and NubotSaif, respectively. Also, Alhamdan et al. (2015) mentioned that the Barhi date cultivar was firm, with a high average hardness value attribute of 119.48 N.

The problem addressed by such a manuscript is concentrated around the conventional methods that are used to inspect the quality of the date fruits and their inability to meet quality requirements. Ordinarily, the traditional methods of quality checking for the date fruits are done manually, mechanically, or automatically based on their external quality attributes such as size, color, and detection of external blemishes. Contrarily, the inspection of internal quality attributes such as total solids, total soluble solid content, acidity, antioxidants, proteins, and other chemical parameters involves using an array of laboratory instruments based on destructive methods, with high accuracy and highly qualified technicians. Despite these requirements of accuracy, it became clear that the traditional methods of quality and safety inspection usually involves a visual inspection as well as, mechanical and chemical tests. This approach results in many disadvantages, such as being slow, inaccurate, suffering from a scarcity of well-trained and experienced labor, weariness, and the tests are destructive, expensive, and provide inconsistent quality. This description of traditional destructive methods for the quality and safety assessment was confirmed by Pullanagari and Li (2021); Caporaso et al. (2018); Escribano et al. (2017); Syahrir et al. (2009); Zheng et al. (2006). All these disadvantages of the conventional methods have a negative impact on the export market and on competitiveness in addition to being unable to meet the consumer expectations. There is also a real and urgent desire to obtain precise and fast, nondestructive, and cost-effective methods to meet increasing year-round demand in providing markets with fresh high-quality fruits.

In this regard, ElMasry et al. (2007); Lu and Ariana (2002) pointed out that the huge advancements in the digital technology has had a substantial positive impact on the quality of agricultural products as it proved its ability to sort fruits and vegetables based on physical, chemical, and sensory characteristics. This can guarantee various quality levels and thus helping to gain consumer confidence and satisfaction and enhances competitiveness. In recent years, many automatic vision systems have spread globally for sorting and grading fruits based on the visible light band, which characterizes fruits according to external quality attributes like color, size, and freeness of external defects. However, the visible light band cannot be used to inspect internal quality parameters. The priority in the fruit quality field is always focused on enhancing the quality of the final product through conducting a precise quality inspection to extract standard and homogeneous quality attributes. One of the most important of these quality attributes that have recently gained interest is the dry matter content (DMC) of the fruit. This is defined as the ratio of dry/fresh weight of the fruit or the sum of soluble (sugar) and insoluble (starch) carbohydrates, proteins, minerals, cell walls, organic acids, fibers, etc., which accumulate in the fruit during maturity development [Suni et al. (2000)]. DMC, moisture content, total soluble solid content (TSS), firmness, and acidity are even more important internal quality attributes and yet they cannot be determined by vision systems based on the visible light band. Therefore, near-infrared spectroscopy (NIRS) is widely used for nondestructive internal quality attribute assessment like soluble solids, dry matter, acidity, total soluble solids (TSSs), and firmness of various fruits such as cherries, grapes, lime, star fruits, tomatoes, dates, mangoes, and apples [Lu (2001); Escribano et al. (2017); Fairuz Omar, (2013); Huang et al. (2018); Kim et al. (2013); Tavakolian et al. (2013); Cortés et al. (2016); Jha et al. (2014); Nagle et al. (2010); Jha and Garg, (2010); Yande et al. (2008); Alhamdan and Atia (2017)]. However, some studies have indicated that the NIR technique has some flaws due to nonlinearity between the spectral signals and the value of each quality attribute. At the same time, NIRS is unable to measure and capture the internal constituent gradients inside the fruit. This leads to inconsistency and conflict between the predicted and measured quality attributes. Furthermore, the NIRS estimates are unable to determine the information about the spatial distribution of quality parameters which are necessary for a more accurate quality analysis [Ariana et al. (2006); Gowen et al. (2007); ElMasry et al. (2012); Chandrasekaran et al. (2019)].

Therefore, there is a focus on using a hyperspectral imaging (HSI) technique, which combines conventional two-dimensional digital images with spectroscopy to detect spatial and spectral features in different electromagnetic spectrum regions such as ultraviolet and infrared bands [Lorente et al. (2012)]. Moreover, Qin et al. (2013); Pullanagari and Li (2021) confirmed that the HSI technique can obtain both spatial and spectral information from an object simultaneously, thus making it a useful nondestructive method to evaluate single objects like fruits, vegetables, or grains. This hyperspectral fingerprint (spatial and spectral information) acquired using HSI reflects the physical and chemical properties of the regions of interest in the image [Ariana et al. (2006); Gowen et al. (2007); ElMasry et al. (2012); Mahesh et al. (2008); Ravikanth et al. (2015)]. HSI studies proved their feasibility in terms of spatial mapping with spectral responses of inspection quality attributes in fruits and vegetables. Here, the running and measuring processes of the HSI technique are carried out by monitoring and storing the data in different modes of reflectance, transmittance, and interactance for analyzing food quality [Gowen et al. (2007); Lorente et al. (2012); ElMasry et al. (2012); Qin et al. (2013)]. These measurements are: the quantities of moisture content, TSS, soluble solid content (SSC), and firmness in the case of fresh okra fruits [Xuan et al. (2021)], nectarines [Huang et al. (2021)], tomatoes [Liu et al. (2015)], strawberries [ElMasry et al. (2007)], apples [Crichton et al. (2018); Noh et al. (2007)], blueberries [Leiva-Valenzuela et al. (2013)], plums [Li et al. (2018)], sweet cherries [Pullanagari and Li (2021)], bananas [Rajkumar et al. (2012)], pears [Li et al. (2016)], nectarines [Munera et al. (2018); Munera et al. (2017)], mangoes [Jödicke et al. (2020); Pu and Sun (2015)], and peaches [Lu and Peng (2006); Zhu et al. (2016)].

Despite the great importance of date fruits, there is scarceness of studies on monitoring and evaluating date fruit quality using a hyperspectral imaging technique. There is only one study exploring the verification of the potential of hyperspectral imaging techniques to detect the fungal contamination of edible date fruits [Teena et al. (2014)]. The main objective of this article is to explore the potential of hyperspectral imaging in the reflectance mode to predict and measure the moisture content, dry matter content, and firmness of some date cultivars as a primary step in identifying and recording the spectral fingerprint of different date cultivars throughout all ripening stages to predict all quality properties (physical, chemical, and sensory).

## Materials and Methods

### Samples

Fresh date samples from two different date fruit cultivars (Khalas and Sukkari) were obtained from a local market in Riyadh, Saudi Arabia, each at the fully mature stage (Tamr). For each cultivar, 100 samples were randomly collected during the 2020 season. Samples were selected based on good appearance and were freedom from defects and injuries. After collecting the appropriate and optimal samples, they were transported by air freight to the Department of Measurements and Process Control, Faculty of Food Science, Szent István University, Budapest, Hungary. Samples were stored under refrigerated conditions at an air temperature of 4^°^C until analysis. Before starting image acquisition of date samples using a hyperspectral imaging camera, the date samples from each cultivar were numbered from 1 to 100 and then physically characterized in terms of length, width, and thickness using a digital vernier caliper (accuracy ±0.01 mm). The mass of each individual sample was measured using a sensitive balance (accuracy ±0.01 g).

### Hyperspectral Imaging System

**FIGURE 1:**
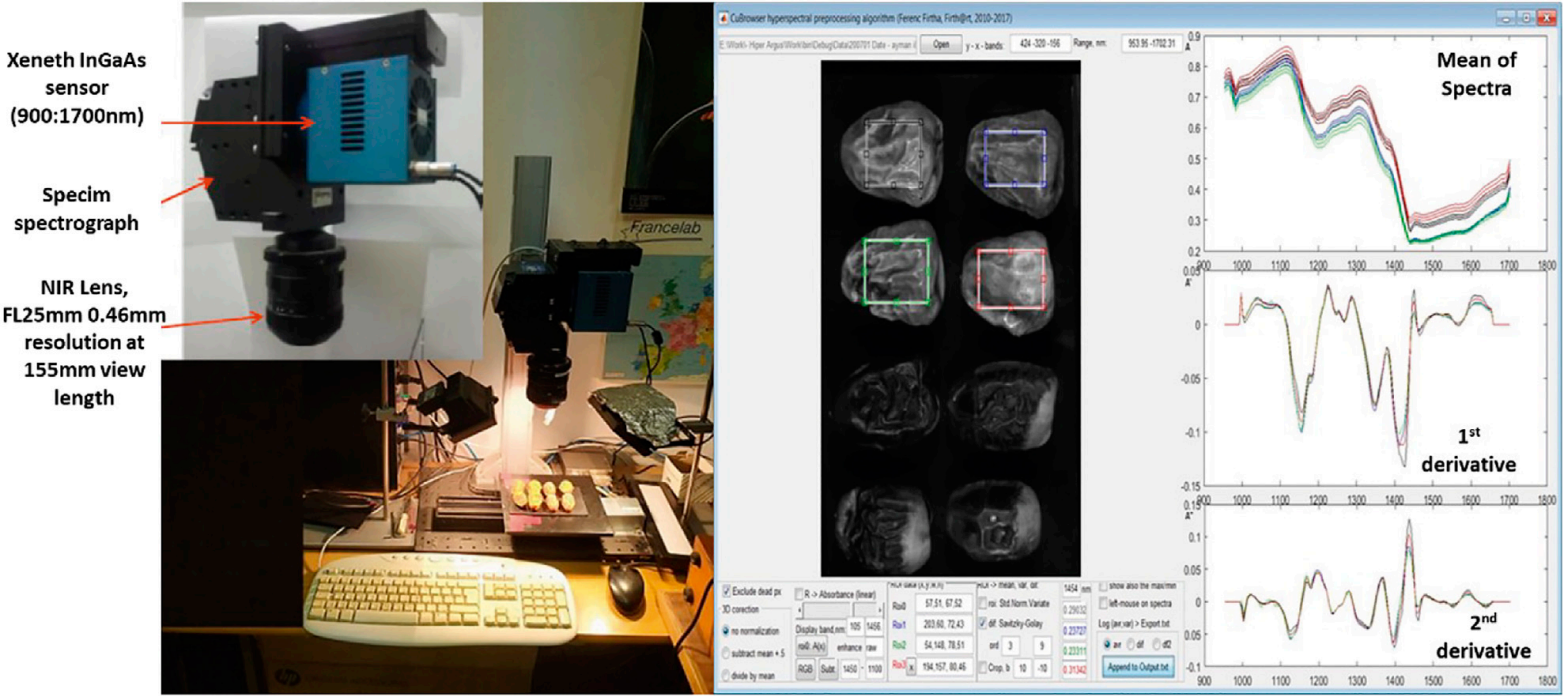
Hyperspectral acquisition system and analysis of spectra of ROI for date samples.

Hyperspectral data were obtained using a HeadWall push broom system (HeadWall Photonics Inc., Fitchburg, MA, United States), with a Canon NIR Lens, an F/2.0 (fast), and a focal lens (FL25 mm) attached, and the lighting system contains two halogen bulb illumination sources with a rating of 2 × 150 W. This power was big enough to gain a proper signal of camera sensitivity, the objective setup, and the slit size of the spectrograph. An optimal illumination geometry of 45/0 (incident angle/observation angle) was applied for ellipsoid objects. Also, a special algorithm (normalization) was used to eliminate the noise of the nonhomogeneous illumination stemming from the uneven surface of the 3D object. A hyperspectral camera was in the spectral range of 900–1700 nm, with a 320 × 256 pixel resolution InGaAs sensor matrix, 14-bit A/D conversion, an 800 nm/156 band = 5.13 nm/px spectral resolution, a 132*100 mm (a 424*320 px length*width) image size, a 100 mm/320 px = 312 µm/px spatial resolution, a 45/0 illumination geometry, and Argus calibration and controlling software [Firtha (2010)]. The stable setup, the proper calibration method, and pixel-noise handling provided the same result for a flat surface as a conventional spectrophotometer. The huge amount of spectral data (1,1 GB) was preprocessed and reduced by the CuBrowser algorithm [Firtha (2010)]. Around a 4′000 px size of the region of interest (ROI) rectangle areas was selected manually on a hypercube inside the date samples, as shown in (Figure 1).

The spectra of ROI pixels were normalized by dividing by their average intensity to overcome inhomogeneous illumination and surface irregularity. The spatial information was used only in this normalization method, which resulted in the average spectrum of ROI having less variance. In the case of uneven surfaces, the proper normalization method of hyperspectral data can provide a much better signal-to-noise ratio than that obtainable with the conventional spectrophotometer method. The raw average spectra and their first and second derivatives were also checked. Numerical differentiation was performed using the Savitzky-Golay algorithm (length: 9 px, order: 3) to eliminate the effects of spectral noise. These derivatives may help to enhance determination efficiency. In this manner, the average spectra of the raw data were considered as the criteria for discrimination between quality attributes for both Khalas and Sukkari date fruit cultivars.

### Reference Measurement Methods

**FIGURE 2:**
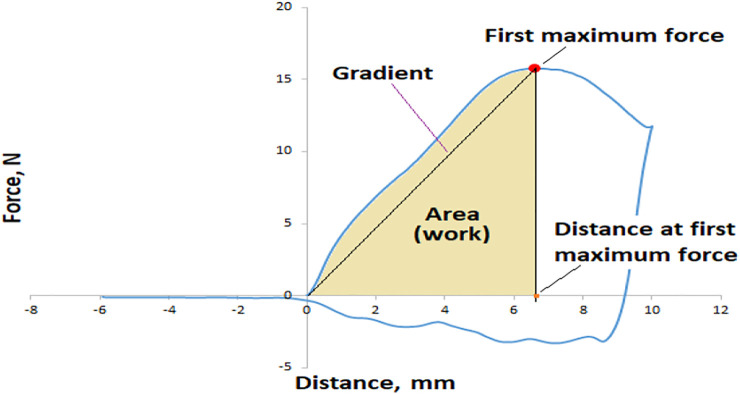
Typical force-deformation curve for date fruit texture analysis.

Reference quality parameters of date fruit samples were analyzed after hyperspectral image acquisition. The date flesh firmness (F) was measured using the TPA test, which was carried out using a texture analyzer instrument TA-XTPlus (Stable Micro Systems, Surrey, UK), equipped with a 5 mm diameter stainless-steel cylinder. The pre-test speed was 1 mm s^−1^, while the test speed during the compression test was 1 mm s^−1^, and the post-test speed was 10 mm s^−1^. The maximum force deformation (expressed in N) was taken as the date fruit firmness and registered on two opposite points in each sample case with a 200 pps (points per second) acquisition data rate. The following parameters were taken into account to explain the firmness of date fruit samples and were determined using an algorithm fracture TPA: the first force peak (first maximum force of rupture in N) and the distance at the first force peak in mm, while the calculated parameters were the gradient (from origo to the first force peak in Nmm^−1^) and the work parameter, which is the area under the force-deformation curve up to the first force peak, defined as the force that acts on an object to cause a displacement (Nmm), as shown in (Figure 2), as described by Szczesniak and Hall (1975); Tanaka (1975) in the application of TPA for solid foods.

Moisture content (MC, %) and dry matter content (DMC, %) were measured for each sample scanned by hyperspectral imaging. Approximately 5 g of the flesh date sample was used. Samples were oven-dried at 105 ± 1°C until the reached a constant mass was obtained according to AOAC (2005). The MC, %, and DMC, %, of the date fruit of each sample were determined in triplicate. A completely randomized design of the experiment was performed. The treatments were randomized to minimize the effects of variation between the two date cultivars. A set of quality attributes was selected for this study, such as size represented by the three dimensions [length (L, mm), width (W, mm), thickness (T, mm), mass (g), MC %, DMC %, the first maximum force of rupture (MF, N), and work (W, Nmm)]. The data were processed for the frequency procedure and an independent *t*-test for groups using the Statistical Package for Social Science (SPSS) software version 20 (Stat Soft, Inc., Chicago, IL, United States ), and a probability value of *p* ≤ 0.05 was considered to show a statistically significant difference among mean values Gardner and Tremblay (2006).

### Analyzing Hyperspectral Data

Hyperspectral reflectance coefficients measured at wavelengths ranging from 900 to 1700 nm were used to develop a prediction model to predict moisture content, dry matter content, and the firmness for date fruits by using the partial least squares regression (PLSR) method. The PLSR process is a qualified method for multivariate regression, that showed great success when applied in spectroscopic studies Nguyen et al. (2006); Viscarra Rossel (2008), Pullanagari and Li (2021). Regression models were performed using software called Unscrambler version 10.3 (CAMO Software AS., Oslo, Norway) to correlate the hyperspectral reflectance data with all quality attributes (moisture content, dry matter content, and firmness) and to provide multivariate calibration. The spectra of all date fruits that underwent hyperspectral scanning were divided into 60% for calibration and 40% for validation, with sets randomly separated. The optimum number of principal components (PCs) was decided based on the little amount of the mean square error of cross-validation for each quality attribute. The accuracy and validity of the calibration and prediction models for moisture content, dry matter content, and firmness quality attributes of date fruits were assessed based on the lowest root-mean-square error of calibration (RMSEC), cross-validation (RMSECV), or prediction (RMSEP), square error of prediction (SEP), and the highest coefficient of determination of calibration and prediction (R^2^_prediction_) through the relevance among both measured and predicted values for each quality attribute. Furthermore, residual predictive deviation (RPD), defined as the percentage among the standard deviations of the reference data and RMSEP, was also used to evaluate the performance of the final prediction model and the bias (b) (average of differences), which is a good indicator of similarity between the calibration and validation sets [Williams (1987); Williams and Sobering (1996); Westad and Martens (2000); Naes et al. (2002); Nicolai et al. (2007); Viscarra Rossel (2008)]. These indicators are formulated as follows:

$$\text{RMSEC}=\sqrt{\frac{1}{{\text{n}}_{\text{c}}}\overset{{\text{n}}_{\text{c}}}{\underset{\text{i}=1}{\sum }}{\left({\hat{\text{y}}}_{\text{i}}-{\text{y}}_{\text{i}}\right)}^{2}}$$

$$\text{RMSEP}=\sqrt{\frac{1}{{\text{n}}_{\text{p}}}\overset{{\text{n}}_{\text{p}}}{\underset{\text{i}=1}{\sum }}{\left({\hat{\text{y}}}_{\text{i}}-{\text{y}}_{\text{i}}\right)}^{2}}$$

$$\text{SEP}=\sqrt{\frac{\sum_{\text{i}=1}^{{\text{n}}_{\text{p}}}{\left({\hat{\text{y}}}_{\text{i}}\;-{\text{y}}_{\text{i}}\;-\text{b}\right)}^{2}}{{\text{n}}_{\text{p}}}}$$

$${\text{R}}^{2}=\frac{\left(\sum_{\text{i}=1}^{\text{n}}{\left(\hat{\text{y}}-\bar{\text{y}}\right)}^{2}\right)}{\left(\sum_{\text{i}=1}^{\text{n}}{\left({\text{y}}_{\text{i}}-\bar{\text{y}}\right)}^{2}\right)}$$

$$RPD=\frac{SD}{RMSEP}$$

$$\text{bais}=\sqrt{\frac{1}{n_{p}}\overset{n_{p}}{\underset{i=1}{\sum }}\left({\hat{y}}_{i}-y_{i}\right)}$$

Here, ${\hat{\text{y}}}_{\text{i}}$ is the predicted value of any quality attribute in date fruit number i, ${\text{y}}_{\text{i}}$ is the measured value of any quality attribute in date fruit number i, ${\text{n}}_{\text{c}}$ is the number of samples in the calibration set, ${\text{n}}_{\text{p}}$ is the number of samples in the prediction set, b is the model bias, $\bar{\text{y}}$ is the mean value for all samples, n is the number of observations in the data set, and SD is the standard deviation of the response variable.

## Results and Discussions

### Date Cultivars Characterization

**FIGURE 3:**
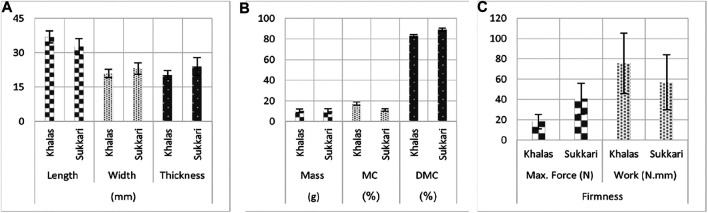
Mean and standard deviation of length, width, and thickness **(A)**; mass, MC, and DMC **(B)**; and firmness attribute **(C)** of Khalas and Sukkari cultivars.

The characterization based on the external quality of the two different date cultivars Khalas and Sukkari revealed clear differences, as shown in (Figure 3A). The majority of the measured physical parameters showed a clear variability within the two date cultivars, for example, the mean and standard deviation of length, width, and thickness were 36.88 ± 2.70, 20.94 ± 1.81, 20.26 ± 1.92 and 32.63 ± 3.47, 23.07 ± 2.54, 24.04 ± 3.9 mm for Khalas and Sukkari, respectively.

Also, Figure 3B shows that Khalas date fruits are moister (17.6 ± 1.58%) compared to the Sukkari cultivar (11.03 ± 1.43%), which showed significant differences (*p* < 0.05) (Table 1). This indicates that Sukkari fruits were harder than the other cultivar. Contrary, the mass parameter did not show any significance within cultivars. The percentage of the DMC property in the Sukkari cultivar date fruits recorded the highest values (88.97 ± 1.43%) as compared to the fruits of the Khalas cultivar (82.84 ± 1.58%). The results of the inspection of firmness for texture of the two different date cultivars are illustrated in Figure 3C and the statistics are found in Table 1. Employing the same approach, the date fruits of the Sukkari cultivar recorded the highest values of the maximum force by mean (40.54 ± 15.19 N) versus 17.94 ± 7.22 N for the dates of the Khalas cultivar. The measures of the DMC and firmness properties obtained for both cultivars showed statistical differences, as mentioned in Table 1. Therefore, the texture of the samples of the Sukkari cultivar was firmer, and this is due to the highest values of DMC and the lowest values of moisture content.

**TABLE 1:** Independent *t*-test for groups of some physical quality parameters of Khalas and Sukkari date cultivars.

Parameters	Group statistics			Levene’s test for equality of variances		*t*-test for equality of means								
	Cultivars		Mean	SD	SE mean	F	Sig.	t	df	Sig. (2-tailed)	Mean Difference	SE Difference	95% confidence interval of the difference	
													Lower	Upper
**L (mm)**	**Khalas**	**Equal variances assumed**	37.87	2.90	0.58	1.19	0.28	5.07	48.00	0.000	3.99	0.79	2.41	5.58
	**Sukarri**	**Equal variances not assumed**	33.89	2.67	0.53	____	____	5.07	47.67	0.000	3.99	0.79	2.41	5.58
**W (mm)**	**Khalas**	**Equal variances assumed**	21.83	1.70	0.34	0.21	0.65	-5.30	48.00	0.000	-2.39	0.45	-3.30	-1.49
	**Sukarri**	**Equal variances not assumed**	24.22	1.49	0.30	____	____	-5.30	47.21	0.000	-2.39	0.45	-3.30	-1.48
**T (mm)**	**Khalas**	**Equal variances assumed**	20.68	1.82	0.36	0.27	0.61	-7.29	48.00	0.000	-3.73	0.51	-4.76	-2.70
	**Sukarri**	**Equal variances not assumed**	24.41	1.80	0.36	____	____	-7.29	48.00	0.000	-3.73	0.51	-4.76	-2.70
**Mass (g)**	**Khalas**	**Equal variances assumed**	10.37	1.26	0.25	10.73	0.00	2.19	48.00	0.034	1.12	0.51	0.09	2.16
	**Sukarri**	**Equal variances not assumed**	9.25	2.24	0.45	____	____	2.19	37.82	0.035	1.12	0.51	0.08	2.16
**DMC (%)**	**Khalas**	**Equal variances assumed**	82.84	1.58	0.32	0.68	0.41	-14.35	48.00	0.000	-6.13	0.43	-6.99	-5.27
	**Sukarri**	**Equal variances not assumed**	88.97	1.43	0.29	____	____	-14.35	47.52	0.000	-6.13	0.43	-6.99	-5.27
**MC (%)**	**Khalas**	**Equal variances assumed**	17.16	1.58	0.32	0.68	0.41	14.35	48.00	0.000	6.13	0.43	5.27	6.99
	**Sukarri**	**Equal variances not assumed**	11.03	1.43	0.29	____	____	14.35	47.52	0.000	6.13	0.43	5.27	6.99
**MF (N)**	**Khalas**	**Equal variances assumed**	18.45	5.76	1.15	10.15	0.00	-8.72	48.00	0.000	-23.29	2.67	-28.66	-17.92
	**Sukarri**	**Equal variances not assumed**	41.74	12.05	2.41	____	____	-8.72	34.44	0.000	-23.29	2.67	-28.72	-17.87
**W (Nmm)**	**Khalas**	**Equal variances assumed**	75.39	25.84	5.17	1.66	0.20	2.85	48.00	0.006	18.56	6.52	5.45	31.66
	**Sukarri**	**Equal variances not assumed**	56.83	19.85	3.97	____	____	2.85	45.00	0.007	18.56	6.52	5.43	31.68

SD, standard division; SE, standard error; L, length; W, width; T, thickness; DMC, dry matter content; MC, moisture content; MF, maximum force; W, work.

### Hyperspectral Analysis

**FIGURE 4:**
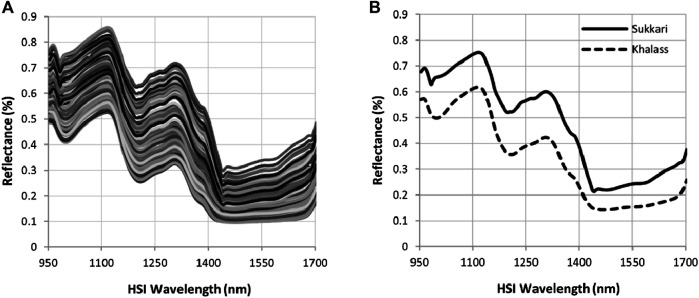
Hyperspectral behavior of date varieties. **(A)** Original hyperspectral wavelength and **(B)** mean of the hyperspectral wavelength of Khalas and Sukkari dates cultivars.

The original spectra in the reflectance mode of all date fruit samples for Khalas and Sukkari cultivars within the spectral range of 950–1700 nm are depicted in Figure 4A. This amount of spectral reflection curves indicates the presence of differences over the hyperspectral imaging range. As is known, the presence of water in the fruit generally leads to a rise in the values of absorption. Hence, the fruits with a higher moisture content have lower values in the reflectance over the applied spectral range. The avereage original spectra recorded for Khalas and Sukkari date fruit samples are shown in Figure 4B. A comparative evaluation of these spectra indicates that they are quite similar.

A considerable difference over the applied spectral range is observed, with reflectance values being higher for the Sukkari cultivar date samples and lower for the Khalas cultivar date samples. This in turn reflects the higher absorbance [log (1/R)] values for the Khalas cultivar date samples and lower absorbance values for the Sukkari cultivar date samples, which means that the Khalas cultivar has a higher number of absorbing bonds. This is evident in Figure 4B, which demonstrates the variation in the average of reflectance spectra for Khalas and Sukkari cultivars which have a similar spectral pattern characterized by having three absorption shoulders focused around 970, 1,110, and 1,300 nm. This confirms the significant differences between the quality characteristics of Khalas and Sukkari cultivars obtained using the reference methods. Therefore, it is critical to recognize the hyperspectral changes associated with physicochemical quality attributes. In the hyperspectral range, the first light shoulder feature was around 970 nm, which is associated with the water absorption shoulder feature due to the second overtone of the O–H stretching [Büning-Pfaue (2003); Lu and Peng (2006); Nicolai et al. (2007); Pullanagari and Li (2021)], which in this hyperspectral region was clearer and more powerful in the Khalas cultivar (Figure 4B). Also, Williams and Norris (2001) confirmed that the spectral shoulder feature around 971 nm is related to strong water absorption, which considers a powerful component in fresh fruits and can be indirectly associated with fruit textural characteristics. Also, Fairuz Omar, (2013) mentioned that the peak around 970 nm is also related to the vibration of the C–H bond present in sugars. However, there was a clear shoulder feature around 1,100 nm, which correlated with the sugar content and hence the ripening stage. These hyperspectral shoulder features corresponded to –H and –OH functional groups, which are related to carbohydrates and water bonds [Osborne et al. (1993); Cordenunsi and Lajolo (1995); Walsh et al. (2004); Rungpichayapichet et al. (2016); Pullanagari and Li (2021)]. Also, this finding agrees with suggestions that most of the relevant information on the SSC concentration is available in the absorption region of the first overtone (800–1,100 nm) and second overtone (1,300–1,600 nm) of water. Kaur (2020) observed that water does not contain sugars, and has the highest peak intensity followed in order by lower peak intensities for the low to high sucrose concentration samples. Also, this result is in agreement with the literature predicting sugars [Bázár et al. (2015)], salts [Gowen et al. (2015)], and honey adulteration detection [Bázár et al. (2016)]. Finally, the decrease observed in the intensity of the reflectance at the spectral peak of 1,300 nm indicates the high absorbance of the water bonds, which was probably due to the effect of O–H groups in water at 1,300 nm. Additionally, in Figure 4B, the other three valleys were related to the strong water absorbance bands occurring at 980, 1,200, and 1,450 nm in the date fruits. This result agreed with Zhu et al. (2017) and Magwaza et al. (2012).

The calibration models for the moisture content (MC), dry matter content (DMC), and firmness (F) of the two date cultivars were implemented by applying partial least squares regression (PLS) using the average spectra of 100 samples of dates. 60% of the total samples were used to create calibration models (calibration set) utilizing the whole spectral range from 950 to 1700 nm, and 40% of the average spectra of samples were used in the validation set. The optimum number of PCs was selected based on the lowest value of RMSECV for each quality attribute. Here, the error recorded the highest values at the beginning and gradually decreased with the increase of the number of PCs in each quality attribute calibration and validation model until its lowest value, when it matched with the best number of PCs. Accordingly, the optimal number of PCs to predict each of MC, DMC, and F (MF and W) was 4, 4, 5, and 4, respectively, as shown in Table 2.

**TABLE 2:** Calibration and cross-validation models for predicting MC, DMC, and firmness in date samples.

Quality attributes		N.	Calibration				Cross-validation			
		PCs	Slope	*R* ^2^	RMSEC	Bias	Slope	*R* ^2^	RMSECV	Bias
Firmness	MC (%)	4	0.92	0.92	0.75	4.77^E−7^	0.91	0.91	0.81	2.95^E−5^
	DMC (%)	4	0.92	0.92	0.75	−1.37^E−6^	0.91	0.91	0.80	−2.92^E−5^
	MF (N)	5	0.91	0.91	3.68	−1.47^E−6^	0.90	0.89	4.1	2.23^E6^
	W (Nmm)	4	0.49	0.49	11.39	5.34^E−7^	0.40	0.40	12.43	2.24^E6^

MC, moisture content; DMC, dry matter content; F, maximum force; W, work; N. PCs, number of principal’s components; *R* ^2^, coefficient of determination; RMSEC, root-mean-square error of calibration; RMSECV, root-mean-square error of cross-validation.

**FIGURE 5:**
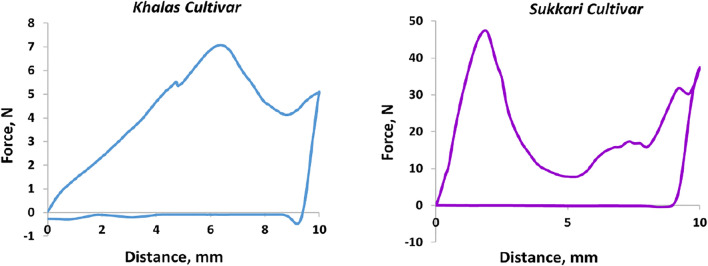
Variation in the force behavior curve among Khalas and Sukkari cultivars.

Furthermore, Table 2 shows that the PLS calibration and cross-validation models were extremely accurate for predicting MC and DMC with the same coefficients of determination (*R* ^2^) of 0.92 and 0.91 for calibration and cross-validation sets, respectively. This accuracy was achieved at the lowest values of RMSEC and RMSECV equal to 0.75 and 0.80% for calibration and cross-validation sets, respectively. However, the accuracy of the calibration and cross-validation models for predicting the firmness attribute varied as there were two criteria for judging the value of firmness (F), which is the first maximum force of rupture (MF) and the work (W). Here, MF (N) achieved the best accuracy of calibration and cross-validation models for the firmness characteristic of date fruits with an *R* ^2^ value of 0.91 and 0.89 for calibration and cross-validation sets, these were accompanied by the lowest error rates of RMSEC and RMSECV of 3.68 and 4.1 N, respectively. This is due to the apparent difference in the first maximum force (MF, N) at which the rupture phenomenon occurs as shown in (Figure 5), which confirms the difference in the texture between the two date cultivars. Furthermore, the amount of DMC and MC is a major reason for the strength of the prediction model and the discrimination between the two types.

However, the work (W, Nmm) criterion for judging the firmness attribute of the dates achieved a lower accuracy for the calibration and cross-validation models. This may be due to the different values of the area under the stress–strain curve at the samples lower in the first maximum force of rupture. This gave results for the measuring values of the work parameter larger for the samples with less firmness compared to the samples with more firmness, which achieved the highest value of the first maximum force at which the rupture phenomenon occurred. This phenomenon can be explained by the elasticity of the cell walls and the moisture content in the cells. As the Sukkari cultivar had a lower moisture content the flesh of its fruit was harder and could have cracked from the effect of the penetration measure head. The Khalas cultivar was juicier due to the higher moisture content, which increased the elasticity of the cells. This finding was in line with an investigation for a study of the variation in the carrot texture under different storage conditions [Kaszab et al. (2009)]. Here, this study showed that there was a very definite reduction in the force/deformation ratio with the decrease of the moisture content of carrots. However, the work ratio considerably increased along with decreasing moisture content during storage. Also, this finding is in correspondence with a study to evaluate the rheological and textural characteristics of black and golden date pastes by Razavi and Karazhiyan (2012), which found that as hardness increases, the total positive area (work) decreases. Here, the value of hardness in the black date pastes was 231.16 ± 22.21 more than those in golden date pastes (210.83 ± 7.57). At the same time, the value of total positive area (work) of black date pastes (5,799.29 ± 593.26) was less than the value of the golden date pastes (6,372.84 ± 350.17).

**FIGURE 6:**
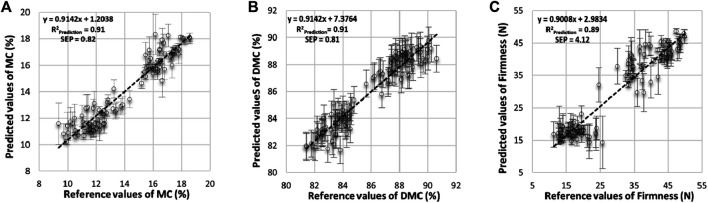
Scatter diagrams of reference vs predicted values for MC, DMC, and firmness (F) accompanied by standard deviation as error bars of predicted values.

Figure 6 depicts the relation between measured values by destructive methods and the corresponding predicted values by the nondestructive method (HSI) for MC, DMC, and firmness [first maximum force (MF) of rupture]. Moreover, Table 3 shows that the prediction model was very accurate for predicting MC and DMC with the same prediction R^2^_Prediction_ of 0.91, with SEPs values of 0.82 and 0.81%, respectively. These results are consistent with successful investigations that focused on both moisture content and dry matter such as in Alhamdan and Atia (2017) and ElMasry et al. (2007), predicting an MC, of 0.94 with an RMSECV of 1.90% and 0.90 with an SEP of 3.874 for date fruits and strawberries, respectively.

**TABLE 3:** PLS prediction models for verification of MC (%), DMC (%), and F (MF, N) in date samples.

Quality attributes	Range	Average	SD	R^2^_Prediction_	SEP	RPD
MC (%)	9.38–17.96	13.59	2.99	0.91	0.82	3.65
DMC (%)	80.04–90.62	86.41	2.99	0.91	0.81	3.69
F (MF, N)	15.54–48.62	32.91	14.11	0.89	4.12	3.42

SD, standard deviation of the validation data set; SEP, standard error of prediction; R^2^_Prediction_, coefficient of prediction; RPD, ratio of the standard deviation to the standard error of prediction.

Also, this finding is in line with a comparative study that used hand-held near-infrared spectrophotometers within the spectral range from 600 to 1,000 nm for dry matter assessment of apples, kiwifruits, and summer fruits. Here, the finding of this study delivered a good prediction model where the values of coefficient of prediction (*r* ^2^) with RMSEP were 0.89 (0.57), 0.92 (0.62), and 0.93 (0.61) for apples, kiwifruits, and summer fruits, respectively [Kaur et al. (2017)]. Also, this result agreed with the results of determination of the DM content of kiwifruit pulp using Fourier-transform near-infrared (FT-NIR) spectroscopy in the NIR range between 1,063 and 1800 nm, where it demonstrated that it was possible to produce excellent PLS regression models for the determination of DM in kiwifruit pulp R^2^_p_, RMSEP, and bias = 91.54, 0.29%, and 0.03, respectively [Kaur (2020)]. Using the same approach, Onsawai and Sirisomboon (2015) achieved *R* ^2^ and RMSECV (0.90 and 3.58%, respectively) for the prediction dry matter content of the pulp durian. Also, Tavakolian et al. (2013) achieved a good correlation between the FT-NIR spectra and the DMC of date fruits with a determination coefficient (*R* ^2^) of 0.94. Also, Clark et al. (2003) got a goodness prediction model where R_p_^2^ and RMSEP were 0.88 and 1.8% between predicted and actual DMCs of Hass’ avocado, respectively. Similarly, the firmness represented in the first maximum force of rupture (MF, N) was predicted with R^2^_Prediction_ of 0.89 and an SEP of 4.12 N resulting from the validation sets. This result is compatible with Zhu et al. (2016), who predicted the firmness attribute of peach pulp as it achieved a correlation coefficient of 0.85 and a residual predictive deviation of 1.74. Also, Leiva-Valenzuela et al. (2013) and Cen et al. (2011) obtained an identical predictive coefficient (*R* ^2^) of 0.87 to predict the blueberry firmness using the HSI technique. Also, Tavakolian et al. (2013) achieved a good correlation between the FT-NIR spectra within spectral range between 833 and 2,500 nm and the firmness of date fruit with a determination coefficient (*R* ^2^) of 0.84 and an SECV of 0.76 N. It is obvious from the values of the coefficient of determination (R^2^_Prediction_, 0.91, 0.91, and 0.89) of prediction that the MC, DMC, and F (MF, N) attributes of date fruits that they can be used for most applications, similarly to as mentioned by Williams (2003) about the values of coefficient of determination of prediction. The fact that the value of *R* ^2^, is between 0.50 and 0.65 indicates that discrimination between high and low values of the measured attribute can be made and can be used for screening and approximate calibration if it ranges from 0.66 to 0.81. Furthermore, if the value was between 0.82 and 0.90, it could be used for most applications, and a value higher than 0.91 is considered to be excellent. However, the second indicator of residual predictive deviation (RPD) used to judge the reliability of prediction models used for date fruit quality attributes [MC, DMC, and F (MF, N)] shows recorded values of 3.65, 3.69, and 3.42 for MC, DMC, and F (MF, N), respectively. Therefore, the hyperspectral imaging technique based on the reference mode has great potency to predict and estimate all MC, DMC, and F (MF, N) attributes for date fruits. This is due to the levels of RPD values corresponding with Saeys et al. (2005), who mentioned that an RPD value of less than 1.5 means very poor predictions and the values ranging from 1.5 to 2.0 indicate that predictions can be used for screening purposes. However, an RPD value lying between 2.0 and 2.5 is suitable for approximate quantitative predictions, an RPD value between 2.5 and 3.0 points out a good model, and the values higher than 3.0 indicate that the prediction performance is considered excellent. It is obvious for the three attributes under study that the validation tests gave nearly similar results to the calibration set, indicating the good performance of the predicting models. From the above, it became clear that the results indicate the feasibility of using the hyperspectral imaging technique for detecting the quality attributes of date fruits. Apart from these properties, it may also be used to monitor and estimate all the other physical, chemical, and sensory properties of date fruits.

## Conclusions

This research presents a preliminary study as a new approach in the field of quality detection of date fruits that uses a hyperspectral imaging technique based on the reflectance mode as a nondestructive and label-free method for estimating some quality attributes such as moisture content, dry matter content, and firmness. The quality detection of two date cultivars using monitoring and the estimation of the three quality attributes, MC, DMC, and F (MF, N), using a successful PLS regression analysis resulted in a powerful prediction model and was classified as a fitting model for most applications. The resulting coefficients of prediction determination (R^2^_Prediction_) were 0.91 and 0.89 for MC, DMC, and F (MF, N), respectively, with the lowest values of SEP of 0.82, 0.81 (%), and 4.12 (N) for MC, DMC, and F (MF, N) over all the captured wavelengths, respectively. These results confirm that the preliminary study for using the NIR hyperspectral imaging technique to monitor and estimate some quality attributes of date fruits, thus especially to assess all MC, DMC, and F (MF, N), was successful. Nevertheless, this technique should be tested more in other date cultivars and on larger samples of fruits in different maturity stages before being disseminated and circulated in the practical form to the quality control systems in the date palm sector. Moreover, this successful preliminary study is considered the first step for a series of investigations to use the hyperspectral imaging technique to inspect the quality of the five maturity stages of date fruits starting from Hababouk, Kimri, Khalal, Rutab, and Tamr. This is to record the spectral fingerprint of the different maturity stages of the fruits of the date and compare it with the physical, chemical, and sensory quality characteristics and to achieve the best prediction model capable of identifying the most important quality characteristics of dates.

## Data Availability

The original contributions presented in the study are included in the article/supplementary material, and further inquiries can be directed to the corresponding author.
